# Impact of Alkaline Pretreatment to Enhance Volatile Fatty Acids (VFAs) Production from Rice Husk

**DOI:** 10.1155/2019/8489747

**Published:** 2019-01-23

**Authors:** Qian Fang, Sinmin Ji, Dingwu Huang, Zhouyue Huang, Zilong Huang, Yunyi Zeng, Yu Liu

**Affiliations:** Department of Municipal Engineering, College of Civil Engineering, Guangzhou University, Guangzhou 510006, China

## Abstract

This study explores the use of alkaline pretreatments to improve the hydrolyzation of rice husks to produce volatile fatty acids (VFAs). The study investigated the effects of reagent concentration and pretreatment time on protein, carbohydrates, and dissolved chemical oxygen demand (SCOD) dissolution after the pretreatment. The optimum alkaline pretreatment conditions were 0.30 g NaOH (g VS)^−1^, with a reaction time of 48 h. The experimental results show that when comparing the total VFA (TVFA) yields from the alkaline-pretreated risk husk with those from the untreated rice husk, over 14 d and 2 d, the maximum value reached 1237.7 and 716.0 mg·L^−1^ with acetic acid and propionic acid and with acetic acid and butyric acid, respectively. After the alkaline pretreatment, TVFAs increased by 72.9%; VFA accumulation grew over time. The study found that alkaline pretreatment can improve VFA yields from rice husks and transform butyric acid fermentation into propionic acid fermentation. The study results can provide guidelines to support the comprehensive utilization of rice husk and waste treatment.

## 1. Introduction

Rice is one of the world's largest crops. According to the survey [1], as much as 660 million tons of rice are cultivated annually worldwide; an additional 800 million tons of agricultural residues (mainly straw) are generated during this process, including more than 113 million tons of rice husk [2, 3]. More than 88% of the world's rice-growing areas are located in Asia. China's share of rice production is as high as 28%; the annual production of rice husk waste is as high as 41.2 million tons. India is the second highest in production at 30.9 million tons [4, 5].

Disposal methods for rice husk in Asian countries and regions are currently limited to feed production and incineration. For example, in the rural areas of Vietnam, as much as 70% of the rice husk is incinerated; India incinerates 23% [6]. Toxic and harmful gases, including carbon dioxide (CO_2_), produced from incineration pollute the environment and directly threaten human health [7]. However, rice husk is also a potential energy resource. Studies have shown that rice husk contains a large amount of organic matter; approximately 20% of this is volatile matter and more than 95% is ash content [8, 9]. Therefore, exploring the reuse of rice husk resources is an important research topic.

Anaerobic digestion is an effective technology option for using this renewable energy resource because it can produce high-value products (e.g., volatile fatty acids (VFAs) and ethanol) in an environmentally friendly way [10–12]. VFAs serve as an efficient carbon source in wastewater treatment [13] and can be used as the synthetic material in polyhydroxyalkanoates (PHAs) [14]. Previous studies have mostly focused on VFA production using excess sludge and swill [15] of livestock excrement, and the anaerobic fermentation of urine organic waste. There has been little research on VFA production from rice husk. Therefore, the anaerobic fermentation of rice husk has significant research applications.

Rice husk contains approximately 28.6% cellulose and hemicellulose, 24.4% lignin, and 18.4% extractive matter [16], which have the potential to be converted to simple sugars and subsequently used for the production of different biofuels. Lignin is an amorphous compound consisting of three monomers: p-coumaryl alcohol (H), coniferyl alcohol (G), and sinapyl alcohol (S), which are linked by ether or carbon-carbon bonds [17]. When considered as a raw material for anaerobic VFA production, the rice husk must be pretreated to break down the lignin seal and to expose the cellulose and hemicellulose content for chemical and enzymatic action (Taherzadeh and karimi, 2008). Therefore, a pretreatment process is necessary for the efficient conversion of lignocelluloses for VFA production.

Different pretreatments have been assessed, including ultrasonic treatment [18, 19], thermal treatment [20, 21], acid and alkaline treatment [22, 23], ozone treatment [24, 25], combined treatment [26–28], and so on. Among these methods, alkali treatment is proved to be effective in inhibiting methanogens activity for VFAs accumulation [22, 29]. Moreover, alkali treatment has the advantages of a short treatment time and lower operational costs than other highly effective pretreatments such as thermal and ozone treatment. Further, alkali treatments have high efficiency on lignocelluloses, especially agricultural residues [30].

This study evaluated the efficacy of alkaline pretreatment by analyzing the conversion of carbohydrate, protein, and dissolved chemical oxygen demand (SCOD) after the hydrolysis of pretreated solids. The goals were to determine the optimum quantity of alkali and processing time, explore the optimal alkali treatment conditions for VFA production, and analyze the laws associated with the release of anaerobic hydrolysis substrate. The study provides a theoretical reference for subsequent rice husk anaerobic fermentation projects.

## 2. Materials and Methods

### 2.1. Materials

The rice husk was provided by the Rice Production Company (Guang Zhou, China) and was prepared for compositional analysis by drying the samples at 45°C for 48 h. The residues were first milled and then sieved, resulting in particle sizes of 0.5–1 mm. This particle size was used in all experiments. Next, the samples were homogenized to avoid compositional differences and stored in plastic bags until use.  Table 1 provides the physical and chemical properties of the rice husk samples.

### 2.2. Methods

#### 2.2.1. Pretreatment

**Figure 1:**
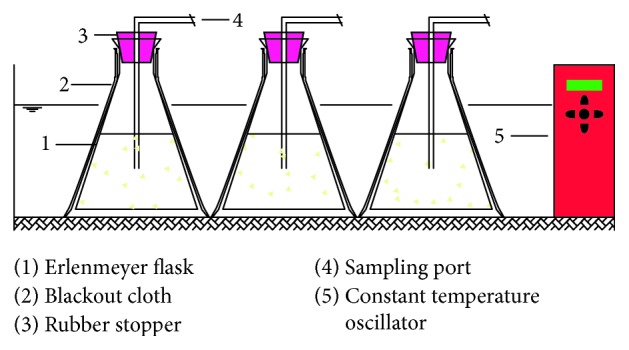
Diagram of pretreatment pilot test facility.

The design of alkaline pretreatment test is shown in Table 2. Alkaline pretreatments were conducted by mixing 9.76 g of rice husk with sodium hydroxide solid (99% w/w), purchased from Zhiyuan Chemical Reagent Co. (Tianjin, China), respectively. The VS concentration was adjusted to 16 g VS·L^−1^ using 500 ml deionized water. After each pretreatment, the slurry was filtered through NEWSTAR filter paper (Hangzhou, China) to separate the solid and liquid. The liquid was collected to determine the total carbohydrates, protein, and SCOD released for each alkali dose and length of pretreatment time (ranging from 6 to 102 hours). The goal was to study the effects of these factors on the subsequent hydrolysis.  Figure 1 shows the alkali pretreatment device. The device consists of a 500 ml Erlenmeyer flask and a rubber stopper with a sampling port. The reactor temperature was automatically controlled in an incubator at 35 ± 1°C. The gas produced during fermentation was collected using the drainage method.

#### 2.2.2. Anaerobic Fermentation

**Figure 2:**
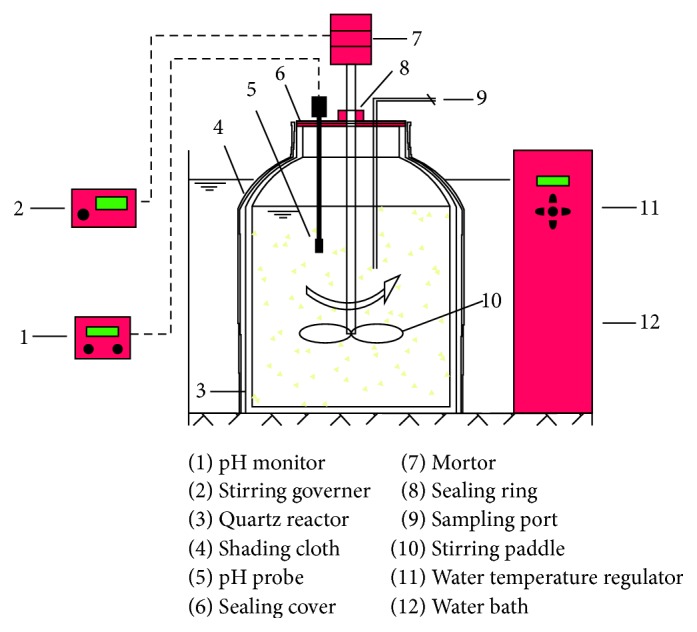
Diagram of the anaerobic hydrolysis acid generator.

The alkali treatment test found that 0.30 g NaOH (g VS)^−1^ generated the optimum alkali level in the rice husk at a treatment time was 48 h.  Figure 2 shows the anaerobic fermentation device used for subsequent experiments. There are two sample sets, labeled 1# and 2#; each included 39.05 g of rice husk and 2 L of deionized water. An adjusted concentration of 0.30 g NaOH (g VS)^−1^ was added to sample 2#. After 48 hours, 10% HCl was added to adjust the pH to 7.0. This eliminated the remaining amount of alkali and initiated the anaerobic fermentation phase to produce VFAs. The fermentation device was placed at a constant temperature in a water bath (35 ± 0.5°C) and stirred at 100 r·min^−1^ for 6:00–8:00 hours everyday for 22 days. No additional fermentation substrate was added, and the fermentation mixture was drained during the run (Table 3).

Figure 2 shows the anaerobic fermentation device. The device is made of quartz glass, and the sealing cover is made of plastic. The inner diameter is 130 mm, the height is 20 mm, and the effective volume is 2 L. The sealing cover has three holes to accommodate the stirring device, the sampling port, and the pH probe. A seal is present between the stirrer and the contact opening of the seal cap. The outer layer of the reactor is wrapped with a blackout cloth. The device was heated to a constant temperature using a water bath; solid-liquid mixing was increased using intermittent stirring with a power agitator.

### 2.3. Analytical Methods

Total solids (TS) and volatile solid (VS) were measured using the gravimetric method [31]. The pH value was measured using a pHS 3C pH meter, and protein was measured using the Coomassie brilliant orchid method [32]. The soluble sugar anthrone [33] and SCOD were measured after the pretreatment by centrifuging the sample at 10000 r·min^−1^ for 10 min. The supernatant was filtered through a 0.45-*μ*m membrane. SCOD was determined using the potassium dichromate method [31].

VFA concentrations (acetic acid, propionic acid, butyrate, and pentanoic acid) were determined using gas chromatography (GC7900 CD-WAX, FID, CNW), with the sample size of 1 *μ*L. The detector temperature and inlet temperature were 250°C and 220°C, respectively. The column temperature was increased from 60°C to 150°C at a rate of 5°C·min^−1^ and was then held for 18 min; the temperature was then increased to 230°C at a rate of 20°C·min^−1^ in 10 min.

## 3. Results and Discussion

### 3.1. Determination of Optimum Conditions for Alkali Pretreatment

#### 3.1.1. The pH Changes in the Process of Pretreatment

**Figure 3:**
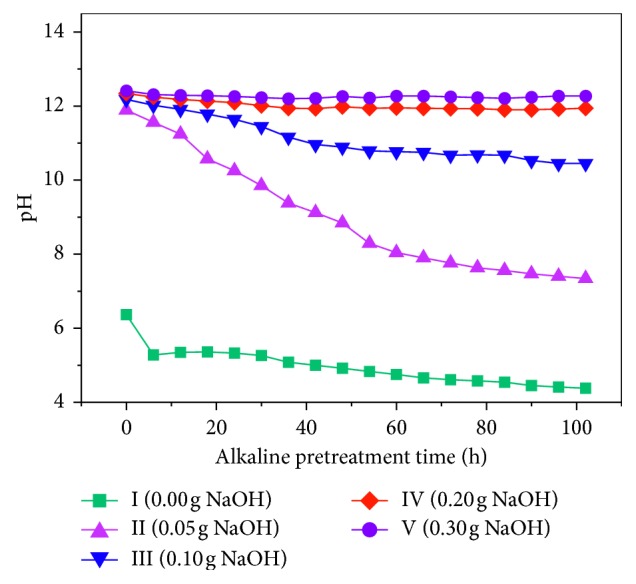
pH curve during alkali pretreatment.

Figure 3 shows the change in the pH value of the rice husk at different alkali pretreatments over time. The initial pH in reactors II, II, IV, and V after the alkali pretreatment was 11.89, 12.18, 12.34, and 12.41, respectively. The greater the amount of alkali added, the slower the pH drop. This is because the higher the alkali content is, the stronger the alkalinity is and the smaller the specific gravity is needed to treat the rice husk. The pH values in reactors II and III began to decline rapidly and then decreased more slowly. The lowest points after 102 hours were 7.34 and 10.45 in reactors II and III, respectively. The pH values in reactors IV and V decreased slowly, stabilizing at 11.95 and 12.25, respectively.

It can be concluded that during the alkaline pretreatment process, the smaller the alkali dosage, the more significant the alkali consumption was. The basic alkali was completely consumed. For the blank group in reactor I, the pH dropped rapidly before 6 h and then decreased more slowly. This may be because at the 0 h point, the sample has just been placed in the shaker and the temperature of the mixture was close to room temperature. After 6 h of heating in the water bath, the temperature rose to 35°C; the rise in temperature led to a drop in pH.

#### 3.1.2. The Protein Changes during Pretreatment

**Figure 4:**
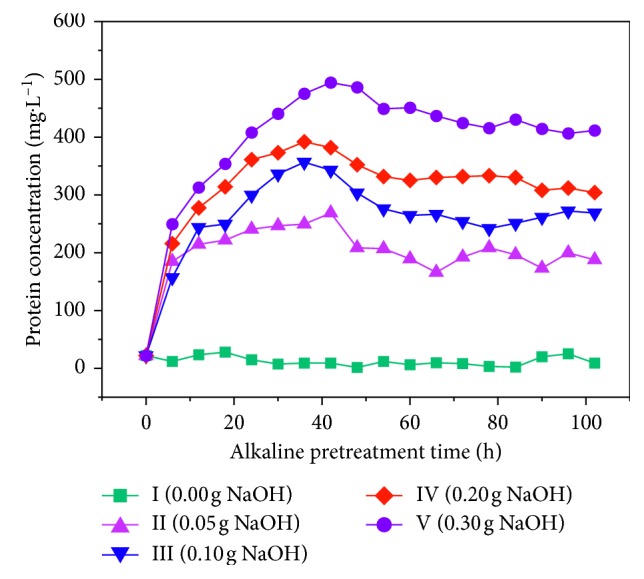
Protein change curve during alkali pretreatment.

Figure 4 shows the changes in rice husk protein when treated with different alkali concentrations. The larger the amount of alkali is, the greater the amount of protein eluted rice husk is. The soluble protein concentrations in reactors II, III, IV, and V increased rapidly before 6 h, then increased more slowly, reached a high point, and then slowly decreased. This is because the amount of alkali in the early stage is sufficient to rapidly dissolve the protein in the rice husk. Once the amount of alkali is reduced, there is a slowly rising trend. As the reaction time increases, the soluble protein levels decrease because the base acts on the protein, denatures the protein, hydrolyzes the polypeptide chain, and finally dissolves into amino acids. It also decomposes under the influence of microorganisms. The eluted amounts of proteins in reactors II, III, IV, and V reached maximum values at 42 h, 42 h, 36 h, and 42 h, respectively, at concentrations of 269, 363, 392, and 505 mg·L^−1^, respectively. A concentration of 0.05 g NaOH (g VS)^−1^ had the best effect in dissolving protein in the rice husk after 42 h of treatment.

#### 3.1.3. The Carbohydrate Changes in the Process of Pretreatment

**Figure 5:**
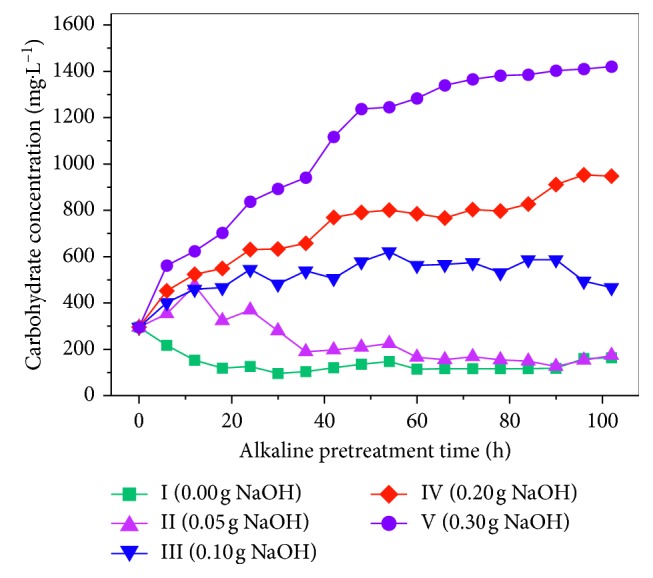
Carbohydrate change curve during alkali pretreatment.

Figure 5 shows the changes in carbohydrate levels in the alkali-pretreated rice husks. It can be concluded that the alkaline-pretreated rice husks have a catalytic effect on the dissolution of carbohydrates in the liquid phase. The greater the amount of alkali added, the greater the amount of dissolved salts. Carbohydrates in reactors III, IV, and V experience an initial rapid increase and then continue to rise. This is because the amount of alkali decreases as treatment time continues. This decreases the ability to destroy the cellulose structure of rice husks, supporting a trend toward rapid and slow carbohydrate dissolution. The carbohydrate levels in reactor II first increased and then decreased. The amount of alkali in the reactor II is low enough that the alkali is quickly consumed. This limits the damage to the cellulose structure. At the same time, microorganisms rapidly decompose the carbohydrates, reducing their concentrations.

Carbohydrate dissolution in reactor V was significantly greater compared to the other alkali treatments. The carbohydrate dissolution rate changed after 48 h; at that point, the carbohydrate elution amount was 1237 mg·L^−1^, which was an increase of 942 mg compared to the initial level of 295 mg·L^−1^. This reflects an increase of three times the initial value. In the reactor II and III treatments, the carbohydrate concentrations were less than 600 mg·L^−1^; the increase was only 1 and 2 times the initial concentration. Therefore, a 0.3 g NaOH (g VS)^−1^ treatment for 48 h is most suitable for the carbohydrate leaching of rice husks.

#### 3.1.4. The SCOD Changes in the Process of Pretreatment

**Figure 6:**
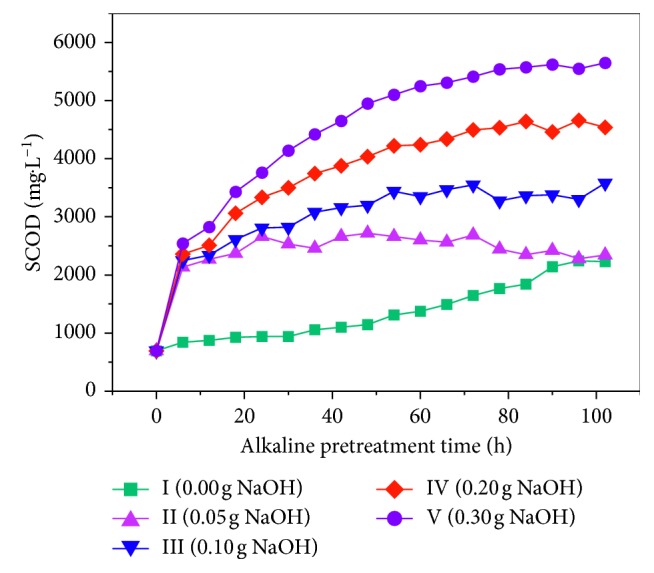
SCOD change curve during alkali pretreatment.

Figure 6 shows the changes in the amount of SCOD released from rice husks when treated with different alkali concentrations. The greater the amount of added alkali, the greater the amount of SCOD released. Within 6 h of adding the alkali, the SCOD dissolution rapidly increased. The SCOD levels in reactors II, III, IV, and V at the 6 h point were 2134, 2246, 2358, and 2540 mg·L^−1^, respectively. These differences were not statistically significant. This may be because the dissolved organic matter in the rice husk dissolves rapidly after the alkali is added. As the treatment time continues, the SCOD dissolution rate in the alkali-treated rice husks gradually slowed; all rates changed near the 48 h point.

The higher the alkali concentration, the faster the SCOD is eluted. The figure shows that the amount of SCOD released from reactor I first increased slowly, then increased rapidly, and finally stabilized after 90 h. At the 90 h point, the SCOD was 2140 mg·L^−1^. When considering the eluted amount of SCOD in reactors II, III, IV, and V at the 6 h point, it appears that the SCOD dissolved within 6 h after the alkali was added was created by the dissolved organic substances in the rice husk itself. Therefore, based on the 2140 mg·L^−1^ level, the SCOD increments of 548, 1060, 1896, and 2810 mg·L^−1^ for reactors II, III, IV, and V at the 48 h point were close to the amount of alkali added. Therefore, a treatment with 0.30 g NaOH (g VS)^−1^ for 48 h is most suitable for dissolving SCOD in rice husks.

In summary, when considering all outcomes discussed above, the results showed that the optimal alkali treatment of rice husk was 0.30 g NaOH (g VS)^−1^ for 48 h.

### 3.2. Effect of Pretreatment on VFAs Production and Various Factors

#### 3.2.1. The Effect of Pretreatment on VFAs Production

**Figure 7:**
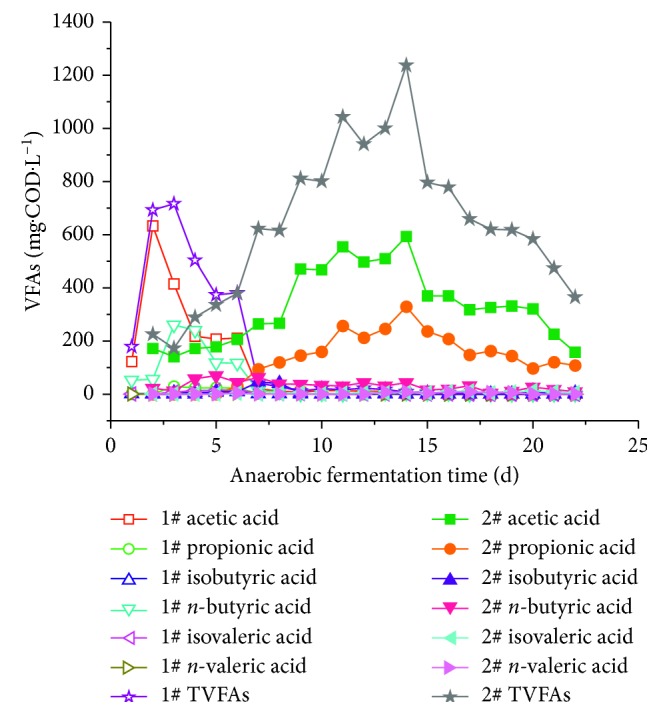
Variation curve of VFA concentrations during anaerobic fermentation.

Figure 7 shows the changes in VFA levels in the hydrolyzate during the alkaline hydrolysis of rice husk. For samples 1# and 2#, the VFAs first increased and then decreased as the test run time continued. The VFA mainly formed from the combined action of anaerobic microorganisms, including hydrolyzed acid-producing bacteria and methanogenic bacteria. When comparing fermentation samples 1# and 2#, there were significant gaps in the timing, incremental changes, and composition of VFS concentrations. The TVFA levels in samples 2# and 1# reach maximum values of 1237.7 and 716.0 mg·L^−1^ on 14 d and 2 d, respectively. When comparing sample 2# with sample 1#, the TVFA concentration increased by 72.9%, and the rate of the increase in VFAs was significantly smaller.

It is likely that during the alkaline pretreatment process, a large amount of organic matter is dissolved into the liquid phase. As such, the VFA accumulation in the anaerobic fermentation of rice husk after alkali treatment is significantly higher compared to the non-pretreated rice husk. During the alkaline treatment, the rice husk carries some anaerobic microorganisms, which are made partially inactive in alkaline conditions. This causes the anaerobic fermentation of organic materials to slow down VFA production. With respect to VFA components, sample 1# consists mainly of acetic acid and n-butyric acid. Sample 2# consists mainly of acetic acid and propionic acid. Samples 1# and 2# represent typical butyric acid-type and propionic acid-type fermentations, respectively. This is likely because after the alkali treatment, the rice husk changes the properties of its fermentation substrate, resulting in changes in V composition of the VFAs.

#### 3.2.2. The Effect of Pretreatment on pH during Anaerobic VFAs Production

**Figure 8:**
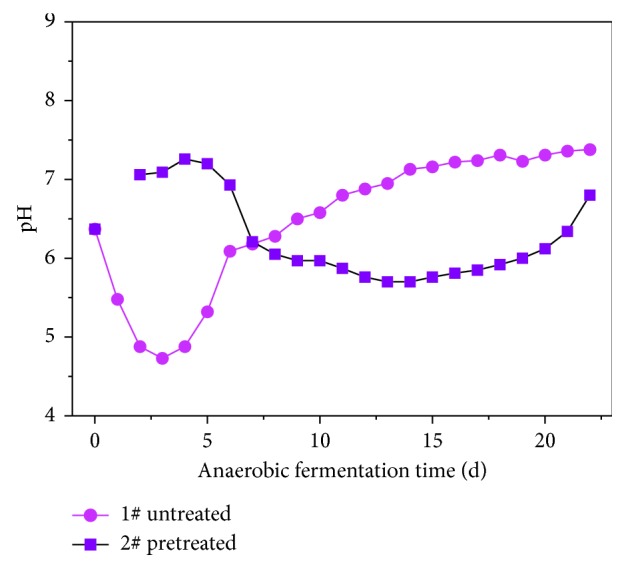
The pH curve during VFA production through anaerobic fermentation.

Figure 8 shows the changes in pH during the anaerobic hydrolysis of rice husk. For sample 1# (untreated), the pH first falls quickly, reaching its lowest point within 3 d. This was followed by a rapid increase and then a slow rise to stabilize after 6 d. Sample 2# (pretreated) began at a stable pH level and then began to decline after 4 d, until 14 d, when there was a rising trend.

Figure 7 shows that pH varies in a similar way as VFAs. Ji Hongfang [34] proposed that changes in pH values during anaerobic fermentation were largely determined by the accumulation of acidic substances, such as VFAs, and the release of NH4^+^-N. Therefore, it is likely that the pH value will decrease due to the conversion of organic matter into acidic substances such as VFAs. As the running time continues, acid accumulates more slowly than the release of NH4+-N and other alkaline substances. This causes the pH to rise. The levels in sample 2# then begin to stabilize, perhaps because HCl is not fully formed after the alkali treatment.

#### 3.2.3. The Effect of Pretreatment on Protein during Anaerobic VFAs Production

**Figure 9:**
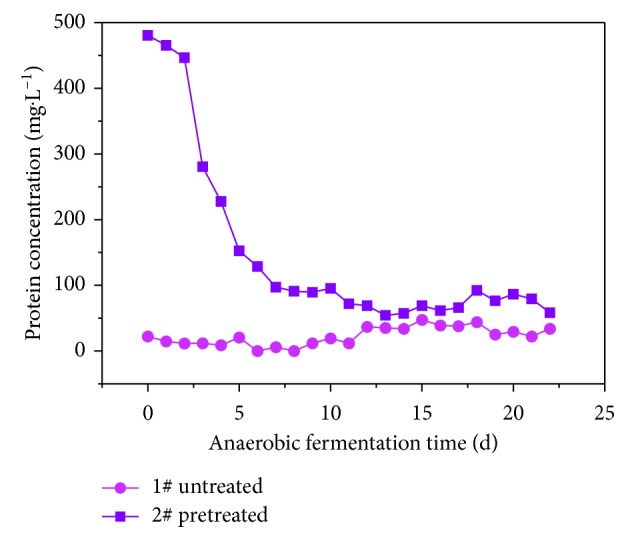
Protein curve during the production of VFAs by anaerobic fermentation.

During anaerobic fermentation, protein decomposes into VFAs. This study analyzed the effects of protein changes on the VFAs of rice husk.  Figure 9 shows the changes in protein levels during the anaerobic fermentation of rice husk with and without alkaline pretreatment. When comparing sample 2# (pretreated) with sample 1# (untreated), the protein concentration in the sample 2# liquid phase is significantly higher, with the maximum value reached in the initial anaerobic fermentation. This is due to the alkaline treatment of the rice husk, which dissolved a large amount of protein and formed soluble protein. For sample 2#, over the test run time, the protein decreased rapidly until the 7 d point, after which the descent slowed.

Protein levels in sample 1# were lower throughout the anaerobic hydrolysis process; protein levels first generally decline and then slowly rise. The protein levels changed due to the hydrolysis effect of anaerobic microorganism on rice husk and the conversion of protease to proteolysis. Figure 7 shows that sample 2# slowly rose in the first 6 d of anaerobic fermentation, while the protein was rapidly degraded. This indicated that the dissolution and degradation of protein in the rice husk was not significantly related to the VFAs during anaerobic fermentation. The protein levels in the rice husk are low, and the VFA levels are small. However, while the protein-VFA decomposition and conversion rate appears to be low, Ji Hong Fang [34] found that compared with carbohydrate wastewater and glycerin wastewater, protein utilization rate and VFAs yield of protein wastewater were the lowest. This may be because of the fact that proteins are biomacromolecules with low hydrolysis efficiency. At the same time, proteins will undergo deamination under anaerobic conditions, and the large amount of free ammonia is highly toxic, which will inhibit the activity of acid-producing microorganisms, resulting in low production of VFAs.

The protein levels in sample 1# increased slowly during the latter stage. This may be due to the release of the anaerobic microorganism enzyme, increasing soluble protein content.

#### 3.2.4. The Effect of Pretreatment on Carbohydrate during Anaerobic VFAs Production

**Figure 10:**
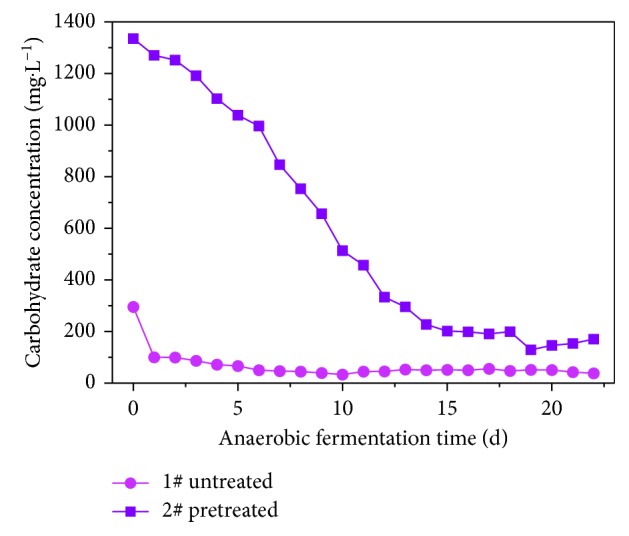
Carbohydrate curve during VFA production by anaerobic fermentation.

Figure 10 shows the changes in carbohydrate levels during the anaerobic water fermentation of rice husk with and without alkaline pretreatment. For sample 2# (pretreated), the carbohydrate level peaked at the initial value and then decreased rapidly until after 14 d, when it stabilized. In sample 1# (untreated), the carbohydrate level began at its maximum value. After 1 d of anaerobic hydrolysis, the carbohydrate level dropped rapidly and then stabilized; the carbohydrates remained significantly higher in sample 2# than in 1# throughout the anaerobic hydrolysis process.

This outcome is due to the changes in the carbohydrates formed by the hydrolysis bacteria in response to carbohydrate dissolution and acidifying bacteria degradation. Figure 7 shows that the degradation rates of the carbohydrates in samples 1# and 2# are very similar to the trends seen with TVFAs. It can be concluded that carbohydrate levels during rice husk fermentation play a leading role in producing VFAs during anaerobic fermentation. During the anaerobic fermentation of rice husk, the acid fermentation was converted from butyric acid to propionic acid. This is because carbohydrate formation in sample 1# is mainly starch, while the carbohydrate in sample 2# is mainly caused by the destruction of the cellulose structure in the rice husk. However, A Cohen [35] and Zoetemeyer et al. [36] found that butyric acid fermentation was the mechanism that produces the acid during the anaerobic fermentation of soluble carbohydrates (such as glucose, lactose, sucrose, and starch). Zhu Guang [37] showed that propionic acid fermentation was responsible for the anaerobic hydrolysis process for cellulose.

#### 3.2.5. The Effect of Pretreatment on SCOD during Anaerobic VFAs Production

**Figure 11:**
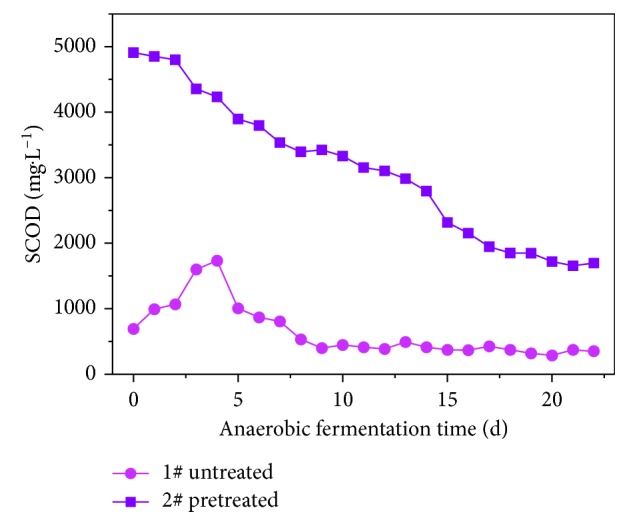
SCOD curve during VFA production using anaerobic fermentation.

In the fermentation broth, the SCOD contains VFAs, carbohydrates, soluble proteins, amino acids, lipids, and humic acids [38].  Figure 11 shows the changes in SCOD in the pretreated rice husk. The SCOD levels were significantly higher in sample 2# (pretreated) than in 1# (untreated). The SCOD in sample 2# reached its maximum level and then began to slow down until after 14 d.

In sample 1#, the SCOD first increased and then decreased. It can be concluded that the results for sample 1# were due to hydrolysis by anaerobic microbes and enzymes. This occurred under the joint action of cell releases inside and outside the organic matter in the liquid phase, combined with the decomposition of organic matter. This led to an initial increase in SCOD, followed by a downward trend.

For sample 2#, after the preprocessing, a large amount of soluble organic matter moved into the liquid phase. As the soluble organic matter in the anaerobic conditions transitioned into the acidification and methane-producing phase, the SCOD gradually declined, until the monosaccharide, VFAs, and other small molecule organic matter were depleted. Then, the SCOD stabilized. As Figure 7 shows, TVFAs dropped rapidly after reaching the maximum value in 14 d. This caused a rapid decline in the SCOD. Compared with sample 1#, TVFA production increased in sample 2# due to the increase in SCOD content.

## 4. Conclusion

1. Different alkali values were used to assess changes in the protein, carbohydrate, and SCOD content of rice husks. The most effective rice husk treatment was 0.3 g NaOH (g VS)^−1^ for a treatment period of 48 h.
2. The alkaline pretreatment effectively increased the amount of VFAs produced by the anaerobic fermentation of rice husks. It also changed the odd and even characteristics of the VFAs produced.
3. During the anaerobic fermentation of rice husks, the effects of carbohydrates and pH were the most significant. The change in pH value may reflect the variations in VFAs.

## Figures and Tables

**Table 1:** Physical and chemical properties of rice husk.

Components	Ash	Moisture content	Total solids	Volatile solids
Content in %	16.4	1.65	98.35	81.95

**Table 2:** Alkaline pretreatment test design.

	I	II	III	IV	V
Rice husk (g)	9.76	9.76	9.76	9.76	9.76
NaOH (g VS)^−1^	0.00	0.05	0.10	0.20	0.30
Deionized water (mL)	500	500	500	500	500

**Table 3:** Design of anaerobic hydrolysis of VFAs.

	1#	2#
Rice husk (g)	39.05	39.05
NaOH (g VS)^−1^	0.00	0.30
Deionized water (L)	2	2

## Data Availability

The data used to support the findings of this study were supplied by the Department of Municipal Engineering under license and so cannot be made freely available.
